# Training on modified model of programme for enhancement of emergency response flood preparedness based on the local wisdom of Jambi community

**DOI:** 10.4102/jamba.v11i1.801

**Published:** 2019-08-20

**Authors:** Andi Subandi, Syahirul Alim, Fitri Haryanti, Yayi S. Prabandari

**Affiliations:** 1 Faculty of Medicine and Health Science, Jambi University, Jambi, Indonesia; 2 Faculty of Medicine, Public Health and Nursing, Gadjah Mada University, Yogyakarta, Indonesia

**Keywords:** programme for enhancement of emergency response, preparedness, training, local wisdom, flood

## Abstract

The community’s role during a disaster within the first 24–72 hours before having government’s aid is crucial in making the condition under control in a timely manner. Disaster management institution, especially at local level, has not had any models for flood preparedness training through participative approach based on the local wisdom of Jambi community to reduce disaster risks. This study tries to evaluate the effectiveness of training programme for flood preparedness based on the local wisdom designed for Jambi community, Indonesia. This research is an experimental study with pre- and post-test control design, in-class training followed by field practice and evaluated using three components: pre-test and post-test evaluations (score scale: 0–100), skill observation during disaster training (score scale: 1–4). The participants consisted of 24 local people chosen from the disaster-prone area; all participated in the disaster training. The score of pre- and post-test evaluations indicates improved post-test result with 71.4 (*p* < 0.005). There was also a skill improvement in the final simulation with 75% excellent score using model modification of programme for enhancement of emergency responses based on the local wisdom. This study completely evaluates the effectiveness of training for flood preparedness based on the local wisdom to improve the knowledge, ability and skill of people in disaster-prone areas.

## Introduction

One of the areas in Jambi Province that has the potential of having annual flood is Muaro Pijoan, Kecamatan Jambi Luar Kota, Jambi, Indonesia. In the 2013 flood incident, two people died. The flood inundated 5348 houses, 40.5 km road, 4 units of public health centres (Puskesmas), 8 units of integrated service centres (Posyandu), 20 schools, 5 units of worship places, 1166 hectares (ha) of rice field (616 ha of it failed to harvest), 132 ha of vegetables farms, 2178 ha of plantations, 215 fish ponds and 11 cattle died (BNPB 2012).

These problems are serious disasters threatening the country’s population, national security, economy and sustainable development. Therefore, to prevent this, it is necessary to have disaster preparedness and mitigation to avoid massive damage and loss in the future (Adelekan 2010; Awuor, Orindi & Adwera 2008). Disaster preparedness that refers to an initiative is intended to enhance the readiness and knowledge between governmental institutions and the community towards a disaster that might happen in certain region. This preparedness focuses on the need to involve the community in mitigation programmes, instead of just expecting them to passively respond to the information source (Eriksen & Prior 2011). To involve the community in preparedness programme, the institutional structure of disaster management agencies, especially at local level, needs to be explicitly made, particularly on their roles and responsibilities. Furthermore, it is necessary to have administrative structures and differentials for complex and controlling command system.

Volunteers from civil defence or local respondents are trained to enhance their skills in managing disaster, in cooperation with local community. It appears that the volunteers have to be paid based on their qualifications and skills. Moreover, the volunteers from civil defence mentioned that the lack of incentives for working at last hours and in emergency situation has become a great challenge. Some local respondents believed that their low number among the community may cause difficulty in doing a timely evacuation to a safe refuge. According to the Indonesian government policy, local and provincial officials are obliged to be at the front line of natural disaster management. While the National Board for Disaster Management (BNPB) and military forces are able to support when needed, however, the policy has not been successful in creating dramatic change at local level. Local Board for Disaster Management (Badan Penanggulangan Bencana Daerah [BPBD]) is planned to be established in all provinces, but only 18 are available at present.

Disaster management institution, particularly at local level, may play important role in effectively reducing the risks of flood with the support from district government and the community; it indicates that even though there are steps taken by these institutions for disaster preparedness, yet these institutions are still facing an obstacle for the implementation at institutional level, and this has created a significant factor to determine effective response from the institutions, including awareness and perception, financial resources, technical resources, effective policy, institutional arrangement, leadership and human resources. The efforts to reduce disaster risk need to be more focused towards the inclusivity of local urban body such as city. Policy-makers need to focus on overcoming the underlying reasons and obstacles hindering effective and efficient preparedness to respond disaster through an appropriate policy mechanism and consultation with all stakeholders. Building awareness and perception among all stakeholders in disaster management supported by community participation may significantly reduce the impact of disaster in the future. The government needs to identify and overcome social and economy problems in the society because these issues may shadow the efforts taken to reduce risk. To obtain an effective reduced risk of flood, it is necessary to develop direct relation between all disaster management institutions, particularly at local level, and the community, with the community leaders as the main focus to ensure these disaster management institutions work complementarily to optimise the preparedness in responding to the flood. Therefore, it is a must for these institutions, especially at local level, to be involved in preparedness programme through participative approach to the community (Harvatt, Petts & Chilvers 2011). Thus, it is necessary to have a training model for community preparedness in disaster-prone areas based on the local wisdom in that region.

This condition of always being affected by floods has made the people of Muaro Pijoan aware of the danger that may occur anytime. However, they cannot relocate to a better and safer place because of many reasons. Some of the reasons are the expensive land price, inability to leave their farm and agricultural land, and attachment to the land owned by their ancestors. Thereafter, the community has had some anticipations in response to the floods that used to come, as follows:

### Building stage house

In the old days, local people had house on stilts (stage house) that have been recognised as traditional houses in many areas in Indonesia. However, because of the progress in time, this kind of stage house is not being used anymore. Some of the causes are modernisation of housings and the difficulty in finding the materials to build stage house.

The best material to build stage house in Jambi is Trembesi, a type of wood that is very hard and has been known hereditarily. Unfortunately, because of the vast land opening and long period of growth, this kind of wood has become rare to find, making it very expensive, way above what the local people can afford of.

Nevertheless, some people have also modified and modernised their houses. Some who can afford it maintain the stage form with concrete columns but still using wood as the material for floor and walls it is because they feel comfortable using wooden flooring and walls even though it is not Trembesi.

However, most people are not using stage house anymore; some build brick walls and cemented or ceramic flooring; the others use wooden materials but not Trembesi. The modernisation of housing is also hindered by the relatively remote location, causing expensive price of materials that eventually forces the local people to adapt with the limitation.

### Boat

As one of the areas where the majority of its people find a living by exploiting the potential of Batang Hari River, it is average for every house to have a boat as their mean of transportation to go fishing in the river. Johan (interview in October 2017) mentioned that they build boats not only to go to the river, but also to anticipate flooding.

### Amben

The long distance to evacuate to higher ground has made Muaro Pijoan residents to choose to stay at their own homes during flood. As a mean to safe their belongings and a place to rest at night, they usually build *amben* as an alternative to solve the problem. *Amben* is a kind of divan for sitting and sleeping and is usually made of wood or bamboo. Johan (interview in September 2017) said that *amben* is commonly used by Muaro Pijoan villagers to keep their things and to sleep at night. Johan mentioned that because the flood in Muaro Pijoan occurs gradually, the local people have time to build *amben*. However, when the flood subsides they throw it away and they will build it again whenever flood occurs. It is certainly inefficient considering flood always hits the area. Apart from the ineffectiveness of that action, nowadays the wooden material to build the *amben* is also difficult to find.

As a result of the expansion of plantations and residential areas, the number of woods in Muaro Pijoan, in fact, has decreased significantly nowadays; therefore, the villagers must certainly find effective solution in using *amben* to anticipate flooding. Johan (interview in September 2017) also stated that at the present time many people are not able to build *amben* properly. The contributing factor is the high number of young people dominating the population in Muaro Pijoan, in which they lack skill in anticipating floods. Even though the villagers have shown mutual cooperation and collaboration in their daily social lives, it is much lesser during flood. It is because of the panic condition among them and their less understanding towards what kind of actions must be taken when flood hits. This underlies the implementation of flood alert training that aims to provide the community with appropriate knowledge and skills to be able to take effective measures when flood occurs.

- Geographically, Desa Pijoan is located on the riverbank of the Batanghari River, which is vulnerable to experience increased water discharge during rainy season. The increased water discharge in the Batanghari River is caused by less sediment area, more buildings at the riverbank and vast deforestation resulting in less water absorption into soil.
- Furthermore, flood in Muaro Pijoan area is also caused by the blocked river flow because of the community’s bad habit of throwing garbage into the river. The community thinks that if the garbage is burnt, it will cause air pollution and bad smell, thus they take the shortcut by throwing the garbage into the river without considering the cause and effect. The blockage caused by sedimentation in the downstream will decrease the ability of the river to accommodate water. Thus, when the rainfall is relatively high, the river is unable to accommodate the high volume of water that is beyond its capacity.
- The people’s lack of knowledge and skills in taking appropriate action when flood hits. Some other information obtained from Mustamim (50 years old) stated that during flood people tend to be panic and unable to do anything except to find higher places such as two-storey house or a tall tree to save themselves from the strong flood current.
- In the social aspect, the people in Desa Pijoan are generally less educated. Based on the statistics from BPS (Badan Pusat Statistik) (National Office of Statistics, Susenas 2014), it indicates that Kabupaten Muaro, Jambi (in which Desa Muaro Pijoan is a part of it) is at the second highest rank of Kabupaten with most uneducated population reaching 230.83%. This relatively low education has resulted in people’s inability to recognise disaster threats in their region. Moreover, their lack of information and knowledge has also contributed in their inability to provide support during flood.
- In the aspect of poor physical condition of buildings in Desa Muaro Pijoan, it is apparent from the majority of houses that do not have the strength to hold the flood. It is indicated by the high number of destroyed houses during the flood in 2013. Furthermore, even though Desa Muaro Pijoan tends to be flooded every year, yet its people show no enthusiasm to build dams around the riverbank. Thus, when the water volume increases it will overflow and inundate their houses.

Since 2009, Indonesia has carried out disaster training using the programme for enhancement of emergency response (PEER) curriculum for Hospital Preparedness for Emergency (HOPE) and Community Action for Disaster Response (CADRE). Community Action for Disaster Response training is one of the training courses given to a community in a disaster area to be able to learn how to deal with disaster in the community. This training contains materials that are very important in disaster management at critical time (first 24–72 h). In the world, the PEER is a regional training programme started in 1998 by the United States (US) Agency for International Development offices for US foreign disaster assistance (USAID/OFDA) to strengthen disaster response capacity in Asia. Stage 3 PEER has been running in nine countries since 2009: Bangladesh, Cambodia, India, Indonesia, Lao People’s Democratic Republic (Laos), Nepal, Pakistan, the Philippines and Vietnam; and it has expanded to Thailand in 2012. The aim of the PEER programme is to increase capacity to manage and prepare for disasters through appropriate training and standards, training courses and curricula that have been developed under the PEER for HOPE and CADRE. Community Action for Disaster Response training is one of the training courses given to a community in a disaster area to be able to learn how to deal with disaster in the community. Therefore, it is appropriate to make imitations of the PEER curriculum, which will later be applied to training programmes for the Murao Pijoan community, Jambi, Indonesia,

## Research methods and design

This study uses the technique of qualitative data analysis to observe the problems faced by the community in overcoming flood, and both qualitative and quantitative data analysis to observe the results of flood preparedness training joined by the community members. The technique of qualitative data analysis is conducted by (1) collecting written verbal data, (2) transcribing spoken verbal data, (3) collecting, selecting and classifying spoken verbal data based on criteria and (4) analysing data and formulating analytical conclusion. The technique of quantitative data analysis is conducted to observe the test results from the questions provided for a certain group. The validity of those questions is obtained by calculating the correlation coefficient of product moment.

Quantitative data analysis is carried out to identify the results of a learning imposed on community group. The process of quantitative data analysis on the two groups includes (1) test results of control group and (2) test results of treatment (experiment) group. After the data are obtained, the test results of the control group and treatment group are tested using *t*-test to obtain a study result, which consists of the advantages and limitations of the learning materials applied. It is intended to compare the post-test results of the two groups (control group and experiment group).

The technique of quantitative data analysis is conducted to identify test results from the questions provided to a certain group. The validity of those questions is obtained by calculating the correlation coefficient of product moment. The quantitative data analysis on the two groups includes (1) test results of control group and (2) test results of treatment (experiment) group. After the data are obtained, the test results of the control group and treatment group are tested using *t*-test to obtain a study result, which consists of the advantages and limitations of the learning materials applied.

To compare the results of posts from both control group and experiment group using the formula below.

### Post-test difference formula in different groups

$$t=\frac{\Sigma x_{1}-\Sigma x_{2}}{\sqrt{\frac{\left(\Sigma x_{1}^{2}-\frac{{\left(\Sigma x_{1}\right)}^{2}}{N_{1}}\right)+\left(\Sigma x_{2}^{2}-\frac{{\left(\Sigma x_{2}\right)}^{2}}{N_{2}}\right)x\left(\frac{1}{N_{1}}+\frac{1}{N_{2}}\right)}{\left(N_{1}-1)+(N_{2}-1\right)}}}$$ [Eqn 1]

The research subjects were the Pijoan village community, Kab. Muaro Jambi was chosen by using a purposive sampling technique to produce 24 people representing several Neighborhood Groups (RT) in Pijoan Village. As for selecting samples or participants in this study through approaches with youth organisations, Chair of Neighborhood Unit (RT) and Pijoan village communities, participants were given action based on stages that included first initial meeting involving village head elements, and youth organisations or community organisations. The aim is to involve various components and provide understanding to them about the importance of implementing a flood disaster preparedness training model to improve community preparedness for disasters. The second is the preparation of the agenda of observation and training activities involving 24 participants whose involvement followed the entire series of research activities.

In this study, the form of the inclusion criteria that will be selected as the research sample is as follows:

Inclusion criteria (1)

- Participants are male and female, aged between 17 and 40 years
- Participants are willing to join the training programme for three meetings (3 days), each of which has a duration of 18 hours
- Participants do not have communication disruptions and can perform well in contraction
- Participants do not have psychiatric disorders
- Participants are willing to be given their identity as long as they are within the scope of their research interests

Furthermore, in this study, exclusion criteria will be determined as individuals who are not recommended to take part in the training programme, while the details are as follows:

Exclusion Criteria (2)

- Individuals are children or adolescents aged under 17 years
- Individuals are children or adolescents who are students (elementary/middle/high school)
- Individuals have problems in terms of communication and have psychiatric disorders
- Individuals have emotional levels that are unstable and difficult to work within a team
- Individuals are not willing to be identified for research needs

### Ethical considerations

Ethical clearance for the study was obtained from Gadjah Mada University (Reference number: KE/FK/233/EC/2017).

## Results

In general, the conclusion of this research indicates that the application of modified model of PEER training based on the local wisdom in Jambi community has been effective in reducing disaster risks in Desa Pijoan, Kabupaten Muaro, Jambi. However, the positive impacts are apparent from the improved knowledge and skills obtained by the community members on the strategy and procedure for disaster preparedness in Desa Muaro Pijoan.

In specific, the conclusions of this study are as follows:

- Increased public knowledge of:
  ■ some dangerous situations and how to respond in those situations
  ■ how to safe their families and to prepare the appropriate response
  ■ first aid and life support
  ■ command system during incident and TRIAGE
  ■ corpse management
  ■ basic techniques of search and rescue
  ■ water and fire emergency

This analysis is used to identify whether there is significant test score results before and after treatment is provided. The analysis is started with descriptive analysis to see the statistics descriptively in the forms of mean, standard deviation, maximum and minimum values. It is followed by normality test, and the final one is comparative test to identify the significant difference of averages.

Table 1 illustrates the descriptive analysis on the test result scores before and after in the experiment group. It is apparent that the average before the treatment is 52.79 with standard deviation of 19.07. After the treatment, the average scores increase to 71.04 with standard deviation of 11.03.

- Improved public skills in the implementation of flood preparedness training to help reduce the risks when flood occurs in Desa Muaro Pijoan. The achievement of maximum scores in the aspect of skills based on the indicators of training materials (see Table 2).
- There is a strong relation between the implementation of disaster preparedness training and the enhanced skills of community members in Desa Muaro Pijoan to reduce the risks of flood incident. This analysis is intended to calculate the correlation between the skill dimensions and the total skill. It means that to obtain information on which dimension has the highest correlation and which dimension has the lowest correlation in shaping one’s skill. This correlation analysis uses the Spearman analysis. (see Table 3).

**TABLE 1:** Before and after test result scores in experiment group.

Test	*N*	Minimum	Maximum	Mean	SD
Pre-test	24	0.00	70.0	52.79	19.07
Post-test	24	50.00	90.0	71.04	11.03

SD, standard deviation.

**TABLE 2:** The achievement of maximum scores in the aspect of skills based on the indicators of training materials.

No	Dimension	Average
1	Hazard review and community organisation	2.88
2	Family safety and readiness	2.63
3	First aid and life support	2.71
4	Incident command system and triage	2.42
5	Water emergency	3.00
6	Basic search and rescue	2.67
7	Corpse management	2.63
8	Final practice simulation	3.75

**TABLE 3:** Dimension correlation.

No	Dimension	Skill	*p*	Remark
1	Hazard review and community organisation	0.217	0.310	Non-significant
2	Family safety and readiness	0.335	0.110	Non-significant
3	First aid and life support	0.228	0.284	Non-significant
4	Incident command system and triage	0.070	0.744	Non-significant
5	Water emergency	0.497	0.014	Significant
6	Basic search and rescue	0.451	0.027	Significant
7	Corpse management	0.356	0.087	Non-significant
8	Final practice simulation	0.071	0.741	Non-significant

The calculation results from Spearman correlation analysis indicate that the correlation coefficient between the shaping dimensions and the skill itself is positive; it means that when these shaping dimensions are increased, the skill will automatically increase as well. The dimensions with the highest correlation are water emergency and basic search and rescue with 0.497 and 0.451 correlation, respectively. These two dimensions do not only have the highest correlation, but they also have significant correlation because the *p*-value is less than 0.05. However, the other dimensions do not have significant correlation towards skill shaping. Not significant does not necessarily mean not having any correlation at all; there is correlation but it is not significant because the *p*-value is above 0.05.

- There are different increase levels of knowledge and skills between PEER group and control group. This analysis is used to identify any differences in the test results after providing treatment in each group. The expected result is no significant difference in the pre-test condition between the two groups.

Based on Table 2, the results of comparative test indicate that the pre-test scores have significant difference between experiment group and control group (*p* < 0.05). Therefore, the hypothesis stating that there is significant difference between the post-test results between experiment group and control group is acceptable.

**TABLE 4:** Comparative test on post-test results.

Group	Mean	SD	*P*	Conclusion
Experiment (post-test)	71.04	11.03	0.000*	Difference exists
Control (post-test)	54.79	9.61		

SD, standard deviation.

* , Independent *t*-test.

## Discussions

The discussions in this research will describe the study results to answer some hypotheses that have been discussed in the ‘Introduction’ section, which are: (1) How is the difference between community knowledge about flood preparedness before and after the training, (2) How is the difference between community skill on flood preparedness before and after the training, (3) How is the relation between community skill on flood preparedness before and after the training and (4) significant difference of post-test results between control group and experiment group. Furthermore, explanation on this discussion will refer to the training results that consist of reaction and learning (Kirkpatrick & Kirkpatrick 2006) as follows.

### People reaction towards flood preparedness training

This stage is one of the evaluation steps to review how the experiment group reacts and responds towards the training conducted by the researchers. Training will be considered as effective if the process is satisfying and it provides positive contributions to the participants. It aligns with Kirkpatrick’s theory (1959), stating that evaluation on the reaction is crucial to do because if there is a participant who reacts negatively and does not like the methods on how the training is conducted, then do not expect that participant to be able to understand the materials delivered. Some components used as reference in evaluating reaction are material, instructor and facility.

Based on the results obtained, it can be wholly formulated that 24 participants stated that the training process has been well facilitated, in relation to training attributes, stationery, use of training media, daily consumption and compensation given to the participants. Furthermore, regarding the instructors, 22 participants stated that the instructors have good potential in delivering the materials, firm in-class management and use vocabularies and grammar that are appropriate with the norms applied in the community. However, two participants stated that the class management should be improved. Moreover, in relation to the materials provided, 20 participants stated that the materials given are very appropriate and it contains important information that they have never known on what to do during a disaster, specifically flood. However, five other participants mentioned that the materials given should be simplified. All these reactions have been reviewed by the researchers based on training reflection addressed after the completion of the training.

### Learning component in the scope of flood preparedness training

This evaluation on the learning aspect still refers to the theory of Kirkpatrick (1959) to review how far the training materials given can be understood, internalised and remembered by the training participants. The domains of competency that will be formulated are the aspects of knowledge and skills; further explanation is as follows.

### Community knowledge of the strategy and procedure of disaster preparedness in Desa Muaro Pijoan

As proclaimed by the government in UU 24 of 2007 regarding disaster management, that before taking a series of action in the management, each community should be educated to clearly identify the efforts and strategies that must be prepared before a disaster occurs. It is an effective step to reduce worst risk caused after the disaster (particularly flood). Before the community faces the real disaster, dissemination of basic knowledge will certainly support their skills in anticipating flood occurrence. It is in accordance with Bandura learning theory (in Blanchard & Thacker 2004), stating that a person who is learning only needs to observe his surroundings to obtain the knowledge of what action should be taken in an organisation.

Trainings that provide materials relating to understanding are always followed by a simulation and personal practice, to allow community members to put the obtained materials into realisation. Some important materials on the understanding of disaster include: (1) identify some potential hazards and how the community responds to those conditions, (2) the action to save family and prepare response to those conditions, (3) first aid and life support, (4) incident command system and TRIAGE, (5) corpse management, (6) basic techniques of search and rescue and (7) water and fire emergency.

After completing all training process in Desa Muaro Pijoan, at the end part the researchers and instructors conducted a final test to observe the achievement of each participant regarding their understanding on disaster knowledge. The test results show that five participants are in ‘Excellent’ category (scores 81–100), and 14 participants are in ‘Good’ category. This indicates that the participants have averagely improved at this stage.

### Community skills on the strategy and procedure of disaster preparedness in Desa Muaro Pijoan

The important thing that should be highlighted as the training result is the community skills in anticipating a disaster incident, particularly flood. Every material prepared by the researchers has been specifically designed to change community behaviour in facing flood occurrence. The structured materials, supported by professional instructors, should be able to change community behaviour towards more positive direction, which aligns with the theory of Ajzen (1991), stating that one’s intention may become the real behaviour when it is under another individual’s control.

The conditioning of a community cannot be separated from the structured system of training design and management. This is in accordance with Purwanto’s opinion (2011), stating that training is an effort to enhance one’s ability and skills towards an experience causing one to obtain new behaviour. Moreover, the training method is to allow an individual to have the ability of doing something, instead of just knowing something.

Align with the process of disaster alert training conducted by the researchers, the results show positive outcomes in every session. Some materials delivered on the first day of training include classification of hazards and consequences, service for community responders, cardiopulmonary resuscitation (CPR) and basic life support (BLS) service. Based on the training conducted on the first meeting, it can be concluded that each participant in general can do training simulation on first aid, including wound treatment, splinting, extrication and transportation of victims.

On the second day, it is apparent that all participants have been able to follow the training process excellently. It is indicated by the high motivation of participants during simulation process provided by the instructors. Based on the training given on the second day, it can be concluded that training participants are generally able to initiate command system by looking for some victims in the simulation of disaster. In conditioning the corpses, it is apparent that all participants have been provided with some materials, such as corpse numbering procedure, cleaning the corpse and taking pictures for documentation. Furthermore, the participants’ skills and ability can be observed in the fire simulation conducted by the researchers. Several techniques to extinguish fire conducted in this simulation are by using portable fire extinguisher, bucket brigade and wet sack.

Furthermore, to increase the practical ability of training participants in anticipating flood, the simulation process is followed by the technique of load lifting to rescue a victim pinched between hard objects. The last material on skill drilling for training participants is water emergency. Every participant is provided with material on the techniques to rescue drowning victims, shallow water rescue and also effective nodes for making some media to rescue victims. From all the simulations provided, it is apparent that all training participants are able to do it well.

In the third meeting, all participants are involved in the final simulation to observe whether all materials on skills have been applied nicely. In general, all participants have been able to do it excellently. After completing the simulation, the session is followed by the application of local wisdom, which is building *amben* and boat. For *amben*, based on the programme designed by the researchers, it is decided that the *amben* form must align with its effective use, which is knock down *amben*. It is because of the high potential of flood occurrence every year, thus *amben* would be a media that is regularly used by the people of Desa Muaro Pijoan to keep their belongings on higher place and save it from the flood.

The development of boat is based on the characteristics of Desa Muaro Pijoan area which is close to a river and averagely people use boats to go fishing. Nevertheless, not all houses in the region have boats; it is because of the relatively difficult method to build a boat and the type of wood being used to build the boat has become rare to find in Desa Muaro Pijoan. To overcome this problem, the researchers and some members of the instructors’ team have designed an alternative boat made of recycled plastic drums. This should be able to overcome the lack of wooden materials to build a boat and maintain wood capacity in Desa Muaro Pijoan. The use of alternative media towards local media is based on the thought and discussion with local people; it is in accordance with the theory of local wisdom by Aulia and Darmawan (2010) in which the results of local wisdom exploration are a collection and way of thinking that are rooted in the culture of a group and the results of observation within a long period of time. The use of this potentially local wisdom is also inseparable from the people’s decision and way of thinking on something that is considered as important and useful. Therefore, in deciding the alternative development of some media that uses the local wisdom, it is necessary to have a review from the contextual aspect based on the area that becomes the focus of the media development. It aligns with Gloriani’s opinion (2013), stating that the context of local wisdom must also be based on the social values believed by the surrounding community, on what is considered good and bad by the community, to determine this it certainly needs consideration process on the applied values.

### The difference in pre-test and post-test scores between control group and treatment group

As described in the research findings, the two community groups that become the focus of discussion in this study are the control group and treatment group. The control group is guided by the BNPB in delivering the materials and in providing both pre-test and post-test. This is in accordance with the opinion of Krisfida (2013), stating that pre-test and post-test are evaluation instruments to compare the training results of the two groups. The pre-test results inform the initial position of the subject before the research is conducted, or in other words it is their proactive history, while post-test is a test given after a treatment has been provided.

The materials covered in the pre-test and post-test include all aspects given by the BPBD to all participants in control group. The questions are about knowledge of potential disaster and also the basic actions that must be taken by the community.

The tests consist of 20 multiple-choice questions. Moreover, the materials composed in the questions are a series of materials that will also be included in the flood alert training that will be conducted by the researchers for the treatment group. The results obtained by the control group indicate that each participant gets different scores. Averagely, all participants do not get maximum score (in the category of excellent), which means that in this control group there is not a single participant in the category of excellent. In addition, four participants receive good test results. Thirteen participants are in the category of sufficient. In the category of insufficient, there are three participants, and the last category is very insufficient with four participants.

Furthermore, the experiment group is analysed to observe any significant difference on the test results before and after the treatment. The analysis is started with descriptive analysis to view the data in descriptive statistics in the forms of mean, standard deviation, maximum and minimum values. Based on the calculation results, it can be shown that the average score before the treatment is 52.79 with standard deviation of 19.07. After the treatment, the average score increased to 71.04 with standard deviation of 11.03. When observed directly, it can be stated that there is an increase in the test scores before and after treatment is given to the experiment group. However, it is necessary to confirm whether this difference is statistically valuable or meaningful, thus a comparative test analysis should be conducted. The calculation results of comparative test using Wilcoxon test indicate that both before (mean = 52.79 and standard deviation = 19.07) and after (mean = 71.04 and standard deviation = 11.03) the treatment have significant difference because the *p*-value (0.000) is below 0.05. It means that this treatment has impact on the training score results. Therefore, the hypothesis stating that there is significant difference of training results before and after the treatment is given to the experiment group is acceptable.

To prove that the results of the training were very useful, the researchers interviewed several respondents who still owned houses in the village of Muaro Pijoan. They mentioned that even though they have houses with high forms that can help them fight flooding, yet through this training people can find out some techniques of assistance that can be done when floods occur. This is very helpful so that they help others stay safe. Furthermore, some alternatives to using simple local wisdom media such as boats and *ambens* are very helpful for residents in evacuating during floods. Although the training process runs short, yet all the material can certainly be done in the event of a flood and can also be done in anticipation before the floods arrive. The community of Muaro Pijoan Village also has a commitment to form a flood alert team so that any knowledge obtained from training can continue to be developed.

Moreover, analysis is also conducted on the control group to identify any significant difference of test results before and after the treatment. The analysis is started with descriptive analysis to view the data in descriptive statistics in the forms of mean, standard deviation, maximum and minimum values. Based on the calculation results, it can be shown that the average score before the treatment is 46.25 with standard deviation of 16.43. After the treatment, the average score increased to 54.79 with standard deviation of 9.60.

When observed directly, it can be stated that there is an increase in the test scores before and after treatment is given to the control group. However, it is necessary to confirm whether this difference is statistically valuable or meaningful, thus a comparative test analysis should be conducted. The calculation results of comparative test using paired *t*-test indicate that both before (mean = 46.25 and standard deviation = 16.43) and after (mean = 54.79 and standard deviation = 9.60) the treatment have significant difference because the *p*-value is below 0.05. Therefore, the hypothesis stating that there is significant difference of training results before and after the treatment is given to the control group is acceptable.

## Conclusion

In general, the conclusion of this research indicates that the application of modified model of PEER training based on the local wisdom in Jambi community has been effective in reducing disaster risks in Desa Pijoan, Kabupaten Muaro, Jambi. However, the positive impacts are apparent from the improved knowledge and skills obtained by the community members on the strategy and procedure for disaster preparedness in Desa Muaro Pijoan.

- In specific, the conclusions of this study are as follows:
  ■ Increased community knowledge after the training on (1) some dangerous situations and how to respond in those situations, (2) how to safe their families and to prepare the appropriate response, (3) first aid and life support, (4) command system during incident and TRIAGE, (5) corpse management, (6) basic techniques of search and rescue and (7) water and fire emergency supported by post-test results of 71.04 and *p*-value of 0.000.
  ■ The community is able to use emergency instruments to build alternative *amben* and boat as one of the potential local wisdoms in helping to reduce risks during flood occurrence in Desa Muaro Pijoan.
  ■ There is increased skill competency particularly in water emergency skills with dimension correlation of 0.014 and basic search and rescue skills with dimension correlation of 0.027 in the experiment group (community members of Desa Muaro Pijoan) after the completion of disaster alert training using the modified model of PEER.
  ■ There is strong relation between community knowledge and skills after the completion of disaster alert training on the community’s preparedness to reduce flood risks in Desa Muaro Pijoan.
  ■ There is a difference in the enhanced community knowledge and skills between the PEER group and control group.
